# Army Legislation

**Published:** 1896-05

**Authors:** 


*Army legislation.* The *Black Hills Press*, of Meade County, South Dakota, in a favorable comment upon the passage of the present Army bill granting to veterinarians the rank of second lieutenant, cannot understand why they are not granted similar recognition to the members of the Army Medical Service. A point well taken.

